# Inequities in HIV prevention among trans and/or non-binary people: a cross-sectional survey analysis of PrEP and PEP awareness and use in Spain

**DOI:** 10.1186/s12939-025-02574-4

**Published:** 2025-07-07

**Authors:** Sara Moreno-García, Paule González-Recio, Marta Donat, Carlos Iniesta, Cinta Folch, María Alonso-Colón, Juan Miguel Guerras, María José Belza

**Affiliations:** 1 Preventive Medicine Department, Severo Ochoa University Hospital, Leganés, Spain; 2 National Health School, Carlos III Health Institute, Madrid, Spain; 3 CIBER Epidemiology and Public Health (CIBERESP), Madrid, Spain; 4 Interdisciplinary Spanish AIDS Society (SEISIDA), Madrid, Spain; 5 Centre of Biomedical Research for Infectious Diseases (CIBERINFEC), Madrid, Spain; 6 CEEISCAT Centre of Epidemiological Studies of HIV/AIDS and STI of Catalonia (CEEISCAT), Health Department, Generalitat de Catalunya, Badalona, Spain; 7 Germans Trias I Pujol Research Institute (IGTP), Campus Can Ruti, Badalona, Spain; 8 National Epidemiology Centre, Carlos III Health Institute, Madrid, Spain; 9 PhD Program in Biomedical Sciences and Public Health, Madrid, Spain

**Keywords:** HIV prevention, Pre-exposure prophylaxis, Post-exposure prophylaxis, Transgender health, Public health disparities

## Abstract

**Background:**

HIV continues to be a global public health challenge, especially in transgender and non-binary people (TGNB). Despite the high effectiveness of Pre-Exposure Prophylaxis (PrEP) and Post-Exposure Prophylaxis (PEP) in reducing HIV transmission, their implementation in this population is unknown. This study describes PrEP and PEP awareness, knowledge of how to access, and use and identifies the factors associated among the TGNB population living in Spain.

**Methods:**

Data from 1468 participants in Transaludes, a national online survey aimed at TGNB people living in Spain, were analysed. The prevalence of awareness, knowledge of how to access both PrEP and PEP through the public healthcare system, and use of each strategy were estimated. Factors associated with awareness and use were analysed using Poisson regressions with robust variance.

**Results:**

A total of 50.5% were aware of PrEP, 14.7% knew how to access PrEP, 2.8% had used it and 1.6% currently, mostly on a daily regimen. A total of 32.5% were aware of PEP, only 13.2% knew how to access PEP and 1.6% had used it, mostly once. Awareness of both strategies was associated with being a non-binary person assigned male at birth (AMAB) or trans woman, being foreign, having a university degree, having undergone biomedical transition and in the past year, having condomless sex with one partner and having engaged in sex work. Use was associated with being older, living in large cities, and in the past year, having condomless sex with two or more partners, having engaged in sex work, and having an STI diagnosis.

**Conclusions:**

Awareness of PrEP is moderate, while that of PEP is lower; in both cases, there is a high lack of knowledge about how to access them, and use of both strategies is low. Disparities, particularly among certain subgroups, such as trans men, younger TGNB, those with low educational level and those living in smaller cities, reflect structural inequities in access to both strategies. Targeted and tailored strategies, including campaigns to improve awareness and access to these prevention tools are needed to reduce these gaps within TGNB populations and achieve the goals for ending the HIV epidemic.

## Background

HIV remains a major public health challenge at the global level, despite the progress made in prevention and treatment in recent decades [1, 2]. The UNAIDS 95-95-95 2025 emphasizes the need to focus efforts on key populations (KPs) to end the HIV epidemic [3]. These populations face extremely high incidence rates due to structural factors such as social inequalities, stigma and discrimination which increase their vulnerability to HIV infection and make it difficult to access health services [4].

Trans and/or non-binary (TGNB) people are one of the most vulnerable KPs in the face of HIV [5–7]. According to UNAIDS, the estimated prevalence of HIV in the trans population of Western Europe and North America is 7.6% [8]; in particular, the incidence of HIV in trans women is 21 times greater than in the general population [9]. The scant evidence on HIV prevalence in trans men who have sex with men suggests that it may be higher than in the general population but lower than among gay bisexual men and other men who have sex with men (GBMSM) [10, 11]. Despite these findings, information on the burden of HIV infection among TGNB people in the European Union (EU) is very limited. Recently, epidemiological surveillance of HIV in Europe has incorporated information on new infections in trans people based on data provided by seven countries, accounting for 0.8% of new cases in 2023. However, the small number of HIV cases reported in this population (*n* = 206) calls for a cautious interpretation of the results [12].

In the last decade, HIV prevention strategies such as post-exposure prophylaxis (PEP) and pre-exposure prophylaxis (PrEP) have proven to be highly effective in reducing HIV transmission [13–15]. Although international organizations recommend the use of PrEP among KPs [16–18], research on the implementation of PrEP and PEP in TGNB people remains limited and shows low awareness, acceptability, and use compared with the high risk of HIV acquisition [19–22]. Potential barriers, such as inequalities in regional PrEP implementation, diverse health models, the cost of the drug, associated stigma, prioritization of gender transition processes versus HIV prevention, and concerns about potential interactions with hormone treatment, have been described [13, 20, 23–27].

Knowing the estimates on the HIV continuum of care in KPs is essential to assess HIV prevention strategies, as reflected in the Dublin Declaration [28]. In Europe, the most recent data show a considerable increase in the number of PrEP users in recent years, although only 0.9% are TGNB [28]. In Spain, since 2019, PrEP has been included in the public healthcare system indicated for at-risk populations, including trans people. However, despite this inclusion, the uptake of PrEP among trans people remains very low [29].

Most of the studies that have explored the scope of PrEP and PEP in the TGNB community are from United States of America (USA) and Canada, where social, health and political contexts differ significantly from the European context, limiting the applicability of their results [7, 19, 30–33]. In addition, they have focused mainly on exploring the need for HIV prevention in trans women with greater vulnerability. Most of these studies recruit participants who are residents of urban contexts, with a high prevalence of racial identities that have historically been highly discriminated against and consequently, face adverse social conditions such as low socioeconomic status, a higher frequency of housing problems, incarceration and a high percentage of sex workers. Furthermore, there is a scarcity of studies that include a considerable number of trans men and non-binary people and that identify factors associated with awareness and use of both preventive strategies, which makes it difficult to recognize barriers, facilitators, and subpopulations with greater vulnerability.

In this context, the recent *Transaludes* survey, which is based on a large and heterogeneous sample of the TGNB population in Spain, enables examination of health status and access to healthcare services, including HIV prevention strategies. The present study aims to estimate the prevalence of PrEP and PEP awareness and use among TGNB people in Spain and to identify the associated factors.

## Methods

### Sample and recruitment

*Transaludes* is a cross-sectional study aimed to explore the health status and use of healthcare services of TGNB people ≥ 15 years of age living in Spain. An online dissemination campaign was conducted nationally through social networks, LGBTQIA+ community organizations, and the study website. Printed materials were also distributed to a national network of 39 gender-affirming care units within the public health system. The survey was conducted between October 2023 and March 2024 with the participation of 1823 people. For the present analysis, people who were diagnosed with HIV infection (*n* = 28) and those who did not answer the question about the result of the last HIV test (*n* = 327) were excluded.

### Procedures

An electronic questionnaire was designed and structured in the following sections: sociodemographic characteristics, degree of visibility as a TGNB person in different settings, sexual practices, biomedical transition processes undergone, drug use, history of HIV test and outcome, diagnosis of sexually transmitted infections (STIs) and a module of questions on awareness and use of PrEP and PEP. At the beginning of the questionnaire, a definition of a trans person was included, as one whose gender is different from the one assigned at birth and explicitly it included non-binary people, regardless of the desire, intention or history of any legal or medical transition process.

### Dependent variables

The questionnaire defined PrEP as follows: *“HIV pre-exposure prophylaxis or PrEP is a medication taken daily by some HIV-negative people to reduce the risk of becoming infected.”*

**PrEP awareness** was assessed with the following question: *“Did you know about HIV pre-exposure prophylaxis or PrEP?”*. The possible responses were *“Yes/No.”*

**How to access to PrEP through the healthcare system** was assessed with the following question: *“Would you know how to request PrEP (HIV Pre-Exposure Prophylaxis) within the healthcare system?”* with *“Yes/No”* as the response options.

**PrEP use** was assessed with the following question: *“Do you take*, *or have you taken PrEP (HIV Pre-Exposure Prophylaxis)?”*. The possible answers were as follows:

- *“Yes*, *I currently take it daily”.*
- *“Yes*, *I currently take it on demand (e.g.*, *when I’m going to have sex on a weekend)”.*
- *“Yes*, *I have taken it in the past*, *but not currently”.*
- *“No*, *never”.*

The first three were categorized as *“PrEP use”*, whereas the last one was categorized as *“Never”*. Based on the affirmative answers, three types of PrEP use were defined: daily, on-demand and past use.

The PEP questions began with the following definition: *“HIV Post-Exposure Prophylaxis is a medication that HIV-negative people can take after risky contact to decrease the risk of becoming infected.”*

**PEP awareness** was assessed with the following question: “*Did you know about Post-exposure prophylaxis of HIV?”* with *“Yes/No”* as response options.

**How to access to PEP through the healthcare system** was assessed with the following question: *“Would you know how to request PEP (HIV Pre-Exposure Prophylaxis) within the healthcare system?”* with *“Yes/No”* as the response options.

**PEP use** was assessed with the following question, *“Have you ever taken HIV POST-Exposure Prophylaxis?”* with *“Yes/No”* as response options. Those who answered *“Yes”* were asked: *“Approximately how many times have you taken HIV Post-Exposure Prophylaxis?”*. The answers were grouped into: *“Once”*, *“Between two and five times”*, *“Between six and 20 times”* and *“More than 20 times”*.

### Independent variables

Independent variables related to gender identity were included, and classified into three main categories based on the proposed terms: trans men, trans women and non-binary people. The latter population was differentiated into two subgroups according to the sex assigned at birth: non-binary people assigned male at birth (AMAB) and those assigned female at birth (AFAB). Sociodemographic variables such as age, region of birth, size of place of residence and higher educational level achieved were included. In addition, variables such as visibility as a TGNB person across all domains of life and engagement in some biomedical transition processes (hormonal and/or surgical) were included. Variables considered by the international guidelines as high-risk factors for the acquisition of HIV over the past 12 months were incorporated. These include the number of sexual partners with whom they have had condomless vaginal/anal sex, having been engaged in sex work, sexualized drug use and STI diagnosis. The recruitment variable was created with the aim of including it in the model adjustment as detailed below.

### Analysis

A descriptive analysis was conducted to examine the main sociodemographic characteristics, visibility as a TGNB person, medical transition processes undergone and high-risk behaviours over the past 12 months. In addition, the distribution of PrEP regimens and the frequency of PEP use were analysed. The prevalence of PrEP and PEP awareness, knowledge of how to access them through the healthcare system, and their use, were estimated by calculating proportions with their respective 95% confidence intervals (95% CI).

To identify the factors associated with each of the variables of interest, awareness and use of PrEP and PEP, four analyses were performed using Poisson regression models with robust variance estimation. Crude and adjusted prevalence ratios (cPR and aPR) and their 95% CI were calculated. Variables with a significance level of *p* < 0.20 were introduced into the initial multivariate model in the crude analysis. The Akaike Information Criterion (AIC) was used to compare models and select the optimal model through a manual “backwards” elimination procedure. Finally, the optimal model was adjusted for the place of recruitment.

All the statistical analyses were performed using via Stata v. 17 (Statacorp, College Station, TX).

## Results

### Participants characteristics

A total of 1468 people were analysed, the majority recruited mainly through social networks and community entities. Four in ten people identified as non-binary individuals (32.6% assigned female at birth [AFAB] and 9.4% assigned male at birth [AMAB]), while 36.2% identified as trans men and 21.7% as trans women. A total of 54.5% were under 25 years of age. The majority were born in Spain and self-identified as white. One third lived in large cities and another one-third lived in medium-sized cities. Almost half had completed High School or a technical degree, and another third had completed university studies. A majority did not perceive their economic situation as good. Nearly half were students, more than a third had a formal job, and one in ten were unemployed. The most frequent sexual orientation was bisexual, followed by heterosexual (Table 1).

**Table 1:** Sociodemographic characteristics of trans and/or non-binary people living in Spain without HIV infection

	Total (*n* = 1468)	
	*n*	%
**Recruitment**		
Community-based organizations	630	42.9
Social media	568	38.7
Survey website	138	9.4
Gender affirming care units	99	6.7
Other	33	2.2
**Gender identity (combined with sex assigned at birth)**		
Trans men	532	36.2
Trans women	319	21.7
Non-Binary AMAB*	138	9.4
Non-Binary AFAB**	479	32.6
**Age (years)**		
15–17	174	11.9
18–24	626	42.6
25–34	427	29.1
35 and older	241	16.4
**Region of birth**		
Spain	1307	89.3
Latin America	100	6.8
Europe	42	2.9
Other	15	1.0
**Race/ethnicity**		
White	1228	86.2
Latinx	106	7.4
Other***	90	6.3
**Inhabitants in place of residence**		
≥ 500.000	469	32.1
50.000-500.000	567	38.8
10.000–50.000	233	15.9
< 10.000	193	13.2
Education level completed****		
Secondary school or less	299	20.4
High School or technical degree	714	48.6
College degree or more	454	30.9
**Self-perceived economic status**		
Very comfortable/comfortable		
Tight	504	35.3
Bad/Very bad	354	24.8
**Employment status** ^**¥**^		
Student	655	45.9
Employee/self-employed	501	35.1
Unemployed	164	11.5
Informal work	62	4.3
Retirement/medical leave	44	3.1
**Sexual orientations**		
Bisexual	540	37.9
Heterosexual/straight	180	12.6
Asexual spectrum	177	12.4
Pansexual	116	8.1
Queer/cuir	112	7.9
Lesbian	100	7.0
Reappropriated terms (*Marika/Bollera*)*****	100	7.0
Homosexual	63	4.4
Gay	37	2.6

*Non-binary people assigned male at birth. **Non-binary people assigned female at birth. *** Other includes individuals who identified as racialized, Gipsy, Black, Asian, Arab/Maghrebi, Afro-descendant, or preferred not to disclose their racial identity. ^¥^ Over the past 12 months. ****Highest completed level of education. *****Terms that were originally used as slurs but have been redefined and reclaim by the LGBTQIA+ community.

Only one in ten people reported being visible as TGNB across all domains of life. Half of the people had undergone some type of hormonal transition process, and a quarter of the people had undergone some type of surgical transition. Over the past 12 months, 6.0% had engaged in sex work, 16.7% had engaged in condomless sex with two or more partners, one in ten had used drugs in a sexual context and only 3.2% had been diagnosed with an STI. Six out of ten participants had never taken an HIV test (Table 2).

**Table 2:** Visibility, medical and/or surgical procedures of transition undergone, numbers of condomless anal/vaginal sex partners, engaging in sex work, sexualized drugs use, STI diagnosis and history of HIV testing among trans and/or non-binary people living in Spain without HIV infection

	Total (*N* = 1468)	
	*n*	%
Visibility in all domains of life*	134	9.1
**Gender-affirming transition undergone**		
Hormonal	833	56.7
Surgical	360	24.5
**Numbers of condomless anal/vaginal sex partners** ^**¥**^		
None	1017	69.8
One partner	197	13.5
Two o more partners	243	16.7
**Sex work** ^**¥**^		
No	1380	94.0
Yes	88	6.0
**Sexualized drug use** ^**¥**^ **		
No	1299	88.5
Yes	169	11.5
**STI Diagnosis** ^**¥**^ ***		
No	1407	96.8
Yes	46	3.2
**History of HIV testing**		
Never	887	60.4
Last 12 months	378	25.7
More than 12 months	202	13.8

^¥^ Over the past 12 months. *Having disclosed being trans or non-binary to all individuals across all life domains. **Substances used for sexual intercourse or during sexual intercourse such as: poppers, non-prescription tranquilizers, non-prescription opiates, cannabis, cocaine, amphetamine/speed, methamphetamine, ecstasy, gamma-hydroxybutyric acid/GHB, mephedrone, ketamine, hallucinogens, alpha-PVP and/or heroin. *** Sexually transmitted infections including bacterial infections such as syphilis, chlamydia, gonorrhea, and viral infections such as hepatitis A or B, HPV, HPV, genital warts, or Mpox

### Prevalence of PrEP awareness and use

A total of 50.5% (95% CI 48.0–53.1) of people were aware of PrEP, 14.7% (95% CI 12.8–16.5) knew how to access PrEP through the public healthcare system and 2.8% (95% CI 2.0–3.7) reported use.

**Fig. 1:**
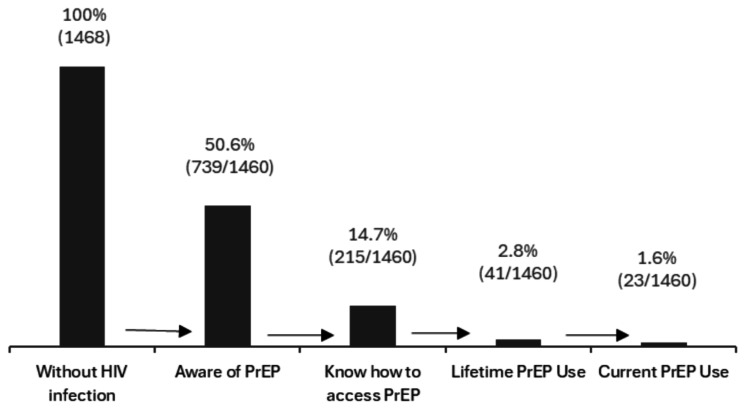
PrEP continuum indicators among the population of trans and/or non-binary people without HIV infection living in Spain (*N* = 1468)

Among total of people 1.2% (95% CI 0.72–1.9) reported past use and 1.6% (95% CI 0.9–2.3) reported current use. Among current users, 91.3% report a daily regime and 8.7% take it on demand (Fig. 1).

### Factors associated with PrEP awareness and use

**PrEP awareness** was significantly associated with being a non-binary person both AFAB and AMAB, being born outside Spain, having university studies, having undergone some biomedical transition process, having had condomless sex with one partner and with two or more partners in the last year, with aPR ranging from 1.0 to 1.7 (Table 3).

**Table 3:** Prevalence and factors associated with PrEP awareness and use among trans and/or non-binary people living in Spain without HIV infection, adjusted for recruitment

	PrEP awareness (*n* = 739)			PrEP use (*n* = 41)		
	Prevalence (%)	aPR	CI95%	Prevalence (%)	aPR	CI95%
**Total**	**50.5**			**2.8**		
**Gender identity (combined with sex assigned at birth)**						
Trans men	43.2	ref.		0.7	ref.	
Trans women	54.0	1.1	0.9–1.2	6.9	2.3	0.7-7.0
Non-Binary AMAB*	71.0	1.4	1.2–1.7	7.2	3.6	1.1–11.4
Non-Binary AFAB **	50.5	1.1	1.0-1.3	1.0	1.1	0.3–3.9
**Age (years)**						
< 30	47.1	-	-	1.3	ref.	
≥ 30	61.5	-	-	7.5	2.9	1.7–5.2
**Region of birth**						
Spain	48.7	ref.		1.8	ref.	
Foreign-born	65.8	1.2	1.1–1.4	11.2	1.7	0.8–3.4
**Inhabitants in place of residence**						
< 500.000	46.1	-	-	1.5	ref.	
≥ 500.000	60.4	-	-	2.1	2.1	1.1–3.9
**Educational level completed**						
< College degree	42.9	ref.		2.4	-	-
College degree	67.8	1.4	1.3–1.6	3.8	-	-
Gender-affirming transition***						
No	47.9	ref.		2.1	-	-
Yes	52.5	1.2	1.0-1.3	3.3	-	-
**Numbers of condomless anal/vaginal sex partners** ^**¥**^						
None	45.0	ref.		1.3	ref.	
One partner	58.7	1.1	1.0-1.3	3.1	1.7	0.7–4.1
Two o more partners	67.9	1.3	1.1–1.4	9.1	2.6	1.3–4.7
**Sex work** ^**¥**^						
No	49.2	-	-	1.8	ref.	
Yes	72.4	-	-	18.4	2.4	1.1–3.9
**Sexualized drug use** ^**¥**^ ****						
No	49.0	-	-	1.8	ref.	
Yes	62.5	-	-	10.7	1.7	0.9–3.3
**STI Diagnosis** ^**¥**^ *****						
No	49.7	-	-	2.0	ref.	
Yes	78.3	-	-	26.0	3.6	2.0-6.7

^¥^ Over the past 12 months. *Non-binary people assigned male at birth. **Non-binary people assigned female at birth. ***Performing a transitional medical procedure such as hormone therapy and/or some type of surgery now or in the past. ****Substances used for sexual intercourse or during sexual intercourse: poppers, non-prescription tranquilizers, non-prescription opiates, cannabis, cocaine, amphetamine/speed, methamphetamine, ecstasy, gamma-hydroxybutyric acid/GHB, mephedrone, ketamine, hallucinogens, alpha-PVP and/or heroin. ***** Sexually transmitted infections including bacterial infections such as syphilis, chlamydia, gonorrhea, and viral infections such as hepatitis A or B, HPV, HPV, genital warts, or Mpox

**PrEP use** was associated with being a non-binary AMAB, being 30 years or older, living in large cities, engaging in behaviours considered risky by clinical guidelines in the past year: having condomless sex with two or more partners, engaging in sex work, and having an STI diagnosis, with aPR varying from 1.1 to 11.7 (Table 3).

### Prevalence of PEP awareness and use

**Fig. 2:**
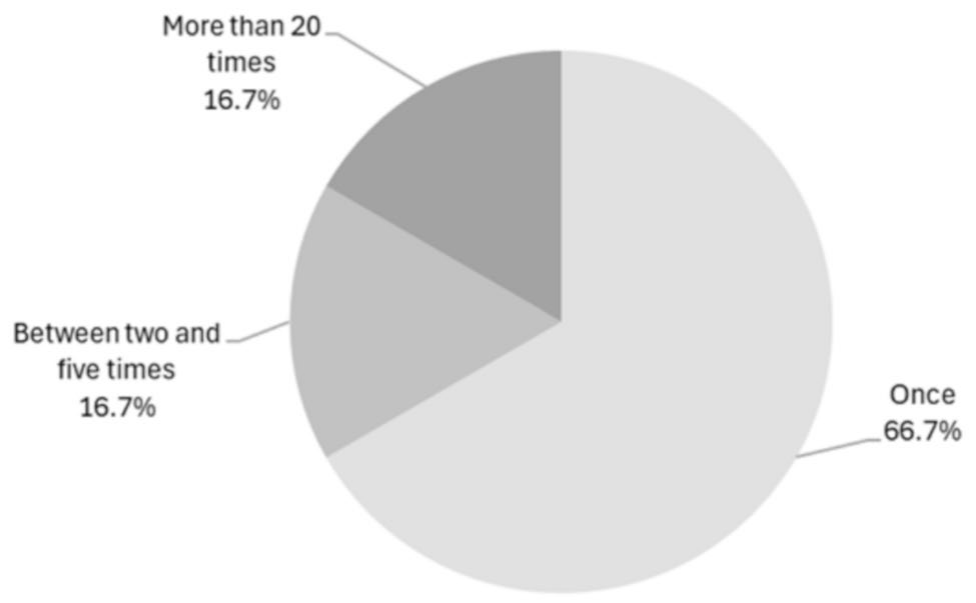
Frequency of PEP lifetime use among trans and/or non-binary people without HIV infection living in Spain (*N* = 24)

A total of 32.5% of the participants were aware of PEP (95% CI: 30.1–34.9), whereas only 13.2% (95% CI: 11.5–15.0) knew how to access it, and only 1.6% reported having used it (95% CI: 1.1–2.4), most of them only once (Fig. 2).

### Factors associated with PEP awareness and use

In the multivariate analysis, **PEP awareness** was independently associated with being a trans woman, a non-binary person AFAB, or a non-binary person AMAB. It was also associated with being 30 years of age or older, being born outside Spain, having a university education, having undergone some biomedical transition process, and engaging in sex work over the past 12 months, with aPR ranging from 1.0 to 1.9. (Table 4).

**Table 4:** Prevalence and factors associated with PEP awareness and use among trans and/or non-binary people living in Spain without HIV infection, adjusted for recruitment

	PEP awareness (*n* = 475)			PEP use (*n* = 24)		
	Prevalence (%)	aPR	CI95%	Prevalence (%)	aPR	CI95%
**Total**	**32.5**			**1.6**		
**Gender identity (combined with sex assigned at birth)**						
Trans men	25.8	ref.		0.4	ref.	
Trans women	40.3	1.2	1.0-1.5	4.4	5.8	1.0-31.1
Non-Binary AMAB*	41.3	1.4	1.1–1.9	3.6	8.6	1.7–42.6
Non-Binary AFAB**	32.2	1.2	1.0-1.4	0.6	1.8	0.2–11.2
**Age (years)**						
< 30	28.5	ref.		0.9	-	-
≥ 30	45	1.2	1.0-1.4	3.9	-	-
**Region of birth**						
Spain	30.6	ref.		0.9	ref.	
Foreign-born	47.8	1.3	1.1–1.6	7.5	3.6	1.3–9.1
**Inhabitants in place of residence**						
< 500.000	28.6	-	-	1.1	-	-
≥ 500.000	41.3	-	-	2.8	-	-
**Education level completed**						
< College degree	27.1	ref.		1.4	-	-
College degree	44.6	1.5	1.3–1.7	2.2	-	-
**Visibility in all domains of life**						
No	7.7	-	-	8.7	ref.	
Yes	12.2	-	-	37.5	2.9	1.1–7.5
Gender-affirming transition***						
No	29.2	ref.		1.0	-	-
Yes	34.9	1.2	1-1.5	2.1	-	-
**Numbers of condomless anal/vaginal sex partners** ^**¥**^						
None	29.1	-	-	1.2	-	-
One partner	36.2	-	-	2.0	-	-
Two o more partners	44.4	-	-	3.3	-	-
**Sex work** ^**¥**^						
No	30.9	ref.		1.2	-	-
Yes	57.5	1.5	1.2–1.9	9.2	-	-
**Sexualized drug use** ^**¥**^ ****						
No	31.3	-	-	1.1	-	-
Yes	41.7	-	-	6.0	-	-
**STI Diagnosis** ^**¥**^ *****						
No	31.9	-	-	1.2	ref.	
Yes	56.5	-	-	15.2	5.9	2.6–13.5

^¥^Over the past 12 months. *Non-binary people assigned male at birth. **Non-binary people assigned female at birth. ***Performing any transitional medical procedure such as hormone therapy and/or some type of surgery now or in the past. ****Substances used for sexual intercourse or during sexual intercourse: poppers, non-prescription tranquilizers, non-prescription opiates, cannabis, cocaine, amphetamine/speed, methamphetamine, ecstasy, gamma-hydroxybutyric acid/GHB, mephedrone, ketamine, hallucinogens, alpha-PVP and/or heroin.*****Sexually transmitted infections including bacterial infections such as syphilis, chlamydia, gonorrhea, and viral infections such as hepatitis A or B, HPV, HPV, genital warts, or Mpox

**PEP use** was associated with being a trans man or non-binary person AMAB, being born outside Spain, being visible as a trans and/or non-binary person across all domains of life, and having a diagnosis of STIs over the past 12 months, with aPR ranging from 1.0 to 42.6 (Table 4).

## Discussion

This study is the first in Spain to estimate the prevalence of PREP and PEP awareness and use in a large sample of TGNB people. Additionally, it aims to characterize PrEP and PEP users. The results highlight a significant opportunity for improvement since, despite the availability of these tools in the public healthcare system, awareness and use remain very low. Only half of the sample was aware of PrEP, one in three was aware of PEP and most did not know how to access these preventive tools.

The prevalence rates of PrEP and PEP awareness and use estimated in our study are consistent with the findings obtained in two European studies. In 2019, a Portuguese study among different KPs reported similar findings regarding the prevalence of PrEP and PEP knowledge and use among trans women [34]. Although it was the first estimate in Europe in this regard, its main limitation was the small number of trans people included and the absence of non-binary individuals. Recent findings from a German study, which included a large sample of trans people with broad representation across different identities, estimated a high prevalence of people with potential PrEP needs (17,3%) [35]. Among them, PrEP awareness was very similar to that observed in the present study (54.1%) but PrEP use was higher (8.1%). However, in the overall sample PrEP use (0.9%) was lower than in our study. In addition, the study did not address the scope of PEP or describe the prevalence of people who know how to access these tools.

Studies conducted outside Europe, mainly in USA and Canada, suggest increased awareness and use of these tools among non-binary AMAB individuals, in line with our findings [32, 33]. However, the levels of awareness and use of PrEP and PEP observed in our study are lower than those reported in other KPs [36], possibly due to the insufficient development of prevention programs aimed at the TGNB population, differences in prevention needs or healthcare barriers resulting from stigmatization and discrimination, among other factors [37]. Concerning PrEP, its progressive implementation in Europe since 2016 has led to disparities in access between countries in the region [28, 38]. The data are consistent with findings from the PrEP Information System (SiPrEP) in Spain, which reports that only 1.4% of users were TGNB, all of which are trans women [29]. Although PrEP was also funded for TGNB in Spain from the beginning of its implementation, this population faces specific barriers such as fear of coming out as TGNB, discomfort when discussing sexual or drug use practices with health professionals, and the stigma of PrEP, among others [23, 26, 39, 40].

Despite its availability in Europe over the past two decades, the extremely low awareness and use of PEP remains striking, a trend also observed in other contexts [22, 41, 42]. Data regarding PEP at other KPs reflect a significant lack of outreach across Europe. For instance, the European MSM Internet Survey in 2017 among MSM (EMIS-2017), found that 44.8% were unaware of PEP and only 4.5% had ever used it [36]. These data also suggest barriers in access, since a third of those who tried to obtain it did not manage to do so [36].

In our study, significant differences between gender identities were identified, particularly in PrEP and PEP use; however, wide confidence intervals warrant caution in interpretation. Indeed, unmet PrEP needs have been described in trans men [43, 44]as well as extremely limited knowledge about HIV transmission and prevention [36]. This disparity may be partially explained by the historical approach of HIV prevention campaigns, which have predominantly targeted cisgender MSM men, reinforcing their position as a primary target population compared to other KPs. This may have favoured gender identities associated with gay culture resulting in greater access to HIV services increases chances of being identified as candidates for PrEP [43].

Given the heterogeneity of sexual practices and orientations within the TGNB population described in this study, HIV prevention and testing programs should focus on sexual practices which would result for better identification of prevention needs across the TGNB spectrum [23, 30, 43, 45–48].

In this study, awareness of, and to a lesser extent, use of, both strategies were found independently associated with certain sociodemographic factors, aligned with the previous research among KPs. These factors include being older, living in large cities and having high education level pointing possible structural barriers [32, 34, 40, 49, 50]. Being born outside Spain was also associated with PrEP and PEP awareness. In fact, most of PrEP users born outside Spain came from countries where PrEP is available for KPs. However, further research is needed due to the health barriers widely described in migrant populations [51]. No association was found with socioeconomic status, consistent with findings from previous studies [31, 33]. More than half of the people in our study had undergone some medical transition process, which was associated with increased knowledge of PrEP and PEP, underscoring the importance of providing integrated care in gender-affirming services [52]. In the present study, only one in ten individuals reported visibility as a TGNB person across all domains of life, which was exclusively associated with PEP use, consistent with previous studies that have identified how being visible as an LGBTQIA+ individual with a healthcare professional contributes to accessing HIV prevention tools [30, 53].

In relation to sexual practices, certain high-risk sexual behaviours that align with PrEP indication criteria in Spain were found associated with awareness and, mostly, with PrEP use [54]. However, in this analysis, these behaviours were not associated with PEP awareness or use, suggesting a gap in the prevention needs of subpopulations of high-risk TGNB subpopulations who could benefit most from this highly effective strategy. Additionally, sexualized drug use was less prevalent in this population than among MSM and was not associated with awareness and use of PrEP or PEP [55, 56].

### Health equity recommendations and implications

This study highlights significant inequalities in access to key HIV prevention strategies, such as PrEP and PEP, among the TGNB population in Spain, exposing the need for public health interventions at multiple levels in Spanish context. On the one hand, as a key population in the HIV epidemic, it is necessary to explore the factors associated with HIV acquisition across the spectrum of TGNB people, their knowledge of HIV transmission, and to research deeper into their specific prevention needs. It is essential to address the inequality stemming from the lack of information on TGNB health at multiple levels of public health and healthcare systems. To this end, gender identity must be incorporated into epidemiological surveillance and health information systems.

The scarcity of large-scale HIV prevention campaigns in Spain over the past decade has had an even greater impact on highly vulnerable populations—such as this one—who, as the results show, face structural and health-related barriers to accessing healthcare services. It is therefore essential to develop specific interventions tailored to the diversity of gender identities, sexual practices, and life trajectories of TGNB people. These efforts must also promote territorial equity and accessibility, particularly for young people and those with lower educational attainment. To this end, improving healthcare professionals’ training in TGNB health and ensuring cultural competence is crucial.

Finally, it is necessary to address the social determinants and axes of inequality that operate on a large scale—particularly the transphobia deeply rooted in the social fabric, which in recent years has become more visible and forceful. Therefore, it is urgent to implement public policies aimed at dismantling and challenging social narratives that reproduce and perpetuate the stigmatization and discrimination of TGNB people, as these have a direct and negative impact on their health.

### Limitations

Our study has several strengths and limitations that should be considered. Although recruitment methods were diversified through various sources with the aim of obtaining a large and heterogeneous sample, including residents of small and rural towns, the findings were obtained from a convenience sample, which limits their generalizability to the entire TGNB population living in Spain. Because participation was voluntary, self-selection bias may have been present. However, online and self-administered data collection tends to result in less underreporting of issues related to sexuality or drug use, minimizing potential social desirability bias [57]. The lower number of trans women, participants with high-risk behaviours or those in situations of greater marginalization may have limited the analyses. Given the low representation of people over 40 years of age, extrapolation to older age groups should be approached with caution. In the multivariable analysis of PrEP and PEP use, wide confidence intervals were obtained, which could be explained by the small size of the user groups. Such imprecision limits the accuracy of the estimates and the robustness of the conclusions; therefore, the results should be interpreted with caution. TGNB individuals participated at all stages of the study, including the research team and throughout all phases of the project. This study was conducted via a depathologizing, destigmatizing and respectful approach, both in the questionnaire design and in the analysis of the results.

## Conclusions

The findings of this study underscore the need to develop public health initiatives focused on improving the awareness and use of PrEP and PEP in the TGNB population in Spain. Achieving these goals requires addressing health inequities and unmet needs particularly among trans men, individuals under 30 years, those without university studies and residents of small and/or rural towns.

Identifying the specific risk factors for HIV acquisition in this population is essential, with a focus on sexual practices and considering the diversity within the TGNB population.

Given the effectiveness of PrEP and PEP and their availability across the European context, it is imperative to investigate barriers to access within this population in order to advance toward the UNAIDS goals for ending the HIV epidemic, ensuring the right to health for all, regardless of gender identity.

## Data Availability

No datasets were generated or analysed during the current study.

## Abbreviations

AFAB: Assigned Female At Birth
AIC: Akaike Information Criterion
AMAB: Assigned Male At Birth
aPR: Adjusted Prevalence Ratio
CI 95%: 95% Confidence Interval
cPR: Crude Prevalence Ratio
EMIS-2017: European Men-Who-Have-Sex-With-Men Internet Survey 2017
EU: European Union
GBHSH: Gay, Bisexual, and other Men who have sex with Men (GBMSM)
KPs: Key Populations
LGBTQIA+: Lesbian, Gay, Bisexual, Trans, Queer, Intersexual, Asexual, and other identities
PEP: Post-Exposture Prophylaxis
PrEP: Pre-Exposure Prophylaxis
TGNB: Trans Gender and/or Non-Binary
UNAIDS: Joint United Nations Programme On HIV/AIDS
USA: United States of America
